# Disparities in expected driving time to opioid treatment and treatment completion: findings from an exploratory study

**DOI:** 10.1186/s12913-022-07886-7

**Published:** 2022-04-11

**Authors:** Abdullah Alibrahim, Jeanne C. Marsh, Hortensia Amaro, Yinfei Kong, Tenie Khachikian, Erick Guerrero

**Affiliations:** 1 Industrial & Management Systems Engineering, College of Engineering & Petroleum, Kuwait University, Kuwait, Kuwait; 2 Geo-Health Lab, Dasman Diabetes Institute, Kuwait, Kuwait; 3 Crown Family School of Social Work, Policy, and Practice, University of Chicago, Chicago, USA; 4 Robert Stempel College Of Public Health and Social Work and Herbert Wertheim College of Medicine, Florida Internation University, Miami, USA; 5 College of Business and Economics, California State University Fullerton, Fullerton, USA; 6 I-Lead Institute, Research to End Healthcare Disparities Corp, Los Angeles, USA

**Keywords:** Opioid use disorder, Medication-assisted treatment, Drive time, Geographic information system, Healthcare outcomes, Disparities

## Abstract

**Background:**

Commuting time to treatment has been shown to affect healthcare outcomes such as engagement and initiation. The purpose of this study is to extend this line of research to investigate the effects of driving time to opioid programs on treatment outcomes.

**Methods:**

We analyzed discharge survey data from 22,587 outpatient opioid use disorder treatment episodes (mainly methadone) in Los Angeles County and estimated the associated driving time to each episode using Google Maps. We used multivariable logistic regressions to examine the association between estimated driving time and odds of treatment completion after adjusting for possible confounders.

**Results:**

Findings show an average driving time of 11.32 min and an average distance of 11.18 km. We observed differences in estimated driving time across age, gender, and socioeconomic status. Young, male, less formally educated, and Medi-Cal-ineligible clients drove longer to treatment. A 10-min drive was associated with a 33% reduction in the completion of methadone treatment plans (*p* < .01).

**Conclusion:**

This systemwide analysis provides novel time estimates of driving-based experiences and a strong relationship with completion rates in methadone treatment. Specifically, the result showing reduced treatment completion rates for drive times longer than 10 min may inform policies regarding the ideal geographic placement of methadone-based treatment programs and service expansion initiatives.

**Supplementary Information:**

The online version contains supplementary material available at 10.1186/s12913-022-07886-7.

## Background

The insidious opioid epidemic affecting the United States has made access to opioid use disorder (OUD) treatment a major target of public health policy. The Centers for Disease Control and Prevention reported a provisional toll of 75,673 opioid-related overdose deaths in the United States in the 12-month period from April 2020–April 2021, accounting for approximately 75% of the record high of 100,306 total drug overdose deaths in that period [1]. Although effective treatment is available in the form of medication for OUDs [2], access issues persist for vulnerable populations, such as racial and ethnic minority groups [3–5]. The most common challenges to accessing OUD medications like methadone and buprenorphine include frequent travel to clinics: often daily, in the case of methadone [6]. Methadone requires far more frequent visits than buprenorphine and is most often prescribed to racial and ethnic minorities, who already face significant barriers to accessing treatment [6]. Therefore, understanding the role of travel time to care in a treatment system that delivers services to one of the most culturally diverse clientele in the United States (Los Angeles, California) is critical to inform effective public health policy.

Studies found that the availability of treatment programs varies widely by region, with some states having many programs within reasonable reach and others—termed “opioid treatment deserts” by Hyder et al.—having very few [7–9]. The standard of reasonable travel distance by car used by Hyder et al. (2021) and others in the field [10] comes from a 2003 study by Beardsley et al., which found that clients who traveled less than 1 mile were found to be 50% more likely to complete substance use disorder (SUD) treatment compared to clients who traveled more than 1 mile, after holding constant demographic variables and type of drug problem [11]. The fact that this study is nearly 20 years old and remains a main measure for reasonable driving distance to SUD care highlights the need for studies to provide updated standards for distance and time based on more recent data. Recent studies that provide newer estimates of travel time to reach OUD treatment by car report median travel times of approximately 9–10 min for large metropolitan areas and approximately 51–56 min for rural areas [12, 13].

Very few studies focused on the association between travel time and SUD treatment outcomes. Studies that found that longer commutes to SUD treatment are generally associated with worse outcomes, from higher rates of treatment dropout and missed doses during the first 90 days of treatment among methadone patients [14] to greater risk of alcohol use a year after inpatient SUD treatment among clients in an unnamed northern California county [15]. Although previous studies have been insightful in establishing the relationship between geospatial access to treatment and outcomes, these studies tended to have smaller samples (2025 clients or less) and overlooked critical commuting elements such as traffic. Rarer still are studies that centered ethnoracial disparities in the context of OUD treatment travel and treatment outcomes.

To bridge this gap, we used existing administrative data to estimate driving time using client ZIP codes and facility addresses. Geographical information systems (GIS) software, Google Maps API, and statistical analysis software provide scalable solutions to estimate the commuting variables for each treatment episode in large administrative or claims datasets. Augmenting such datasets with commuting variables for analyses can leverage the richness of claims or administrative data to address previously unanswerable questions about the role of commuting in opioid treatment outcomes after initiation. The intricacy, urgency, and lack of comprehensive data on ethnoracial disparities in spatial access to SUD care motivated our exploratory study on estimated driving time (EDT) for OUD clients in Los Angeles County, with nationwide implications.

Our aims in this study were two-fold: (1) to examine EDT for subpopulations in Los Angeles County and (2) to quantify the association between travel time and completion of the clients’ treatment plan. We use a combination of GIS geolocating and Google Maps API (a source of road network and traffic data) to calculate EDT and distance, consistent with studies across the health care domain [9, 16–21].

## Methods

### Data

This study involved a retrospective, multiyear, cross-sectional analysis of OUD treatment episodes that accounted for EDT between the clients’ ZIP code and treatment facility. We analyzed discharge survey data from 22,587 OUD treatment episodes in Los Angeles County with client ZIP codes. We relied on client administrative data from the Los Angeles County Participant Reporting System. The data came from a parent study funded by NIDA (R33 DA03563401) that focused on SUD treatment programs that served communities with more than 80% Latino or African American residents in Los Angeles County. The multiyear cross-sectional data included 12,247 clients aged 12 or older served by 125 unique SUD treatment programs. This sample included 96 (76.8%) SUD programs that offered outpatient counseling services to clients with OUD and 32 (25.6%) outpatient programs that offered methadone (Note: some programs offer both). These two types of programs serve more than 95% of all clients entering publicly funded OUD treatment in Los Angeles County. The analysis was conducted at the episode level such that each client’s characteristics and EDT were included. We analyzed two mutually exclusive services: medication-assisted treatment (i.e., methadone) and nonmedication outpatient counseling.

### Geographical variables

We geocoded each client at the population-weighted centroid of their reported ZIP code using ArcGIS Pro (ESRI, 2021). We calculated the population-based centroid of ZIP codes using census block-level population data [22]. We considered using ZIP Code Tabulation Area (ZCTA – a generalized spatial representation of ZIP code service areas). However, we found that using the higher resolution ZIP codes is more precise for geolocating clients and that only 0.59% of study sample episodes would be mapped to larger ZCTA using cross-walk files. Concerns over using ZIP code are more common when creating spatial aggregates. Because our study objectives are to determine the role of travel time on client-level experiences, we expect less problematic experiences with ZIP codes.

EDT via automobile was determined from the ZIP code centroid to the treatment facility using Google Maps Distance Matrix API [23]. We calculated the EDT during mornings (9:30 a.m.) on weekdays. We included traffic in EDT calculations, unlike studies reviewed by Kelly et al., none of which accounted for traffic congestion [20]. Traffic congestion ought to be accounted for when estimating driving time in densely populated metropolises like Los Angeles, where travel time by car varies mainly depending on the time of day. The EDT from the Google Maps Distance Matrix API represents the best-guess estimate which includes historical traffic patterns during the specified date and time. Therefore, the drive-time estimate obtained for each episode includes traffic congestion by default. Additionally, we are able to obtain an optimistic drive-time estimate – or an estimate of driving time with minimal traffic. We use the difference between optimistic driving time and best guess driving time to quantify the proportion of driving time attributable to traffic congestion. We compared the EDT with traffic obtained from Google Maps Distance Matrix API for 100 randomly selected episodes to ESRI road network and traffic [24] to assess the validity of the seimates. Additionally, the single-mode transportation approach of focusing on travel time by car used in this analysis has been found to predict a similar pattern of health care accessibility compared to multimodal approaches, with a high correlation observed between single-mode and multimodal accessibility rates [25].

### Treatment outcome variables for aim 2

The key outcome variable for Aim 2 was treatment plan completion based on six official discharge codes. The first two codes evaluated whether the client completed the treatment or recovery plan or was referred or transferred, whereas the next four codes defined clients who left without making progress, died, got incarcerated, or other discharge status [26]. For the first outcome, we coded participants as 1 if the clinician reported the client completed the treatment or recovery plan for that episode and 0 if not. These measures have been used to evaluate treatment completion in regional [27–29] and national [30–34] studies. They do not include information on the number, type, or description of the treatment plan or its goals.

### Explanatory variables

The independent variables of interest included clients’ self-reported sex, measured as a dichotomous variable (1 = female, 0 = male). The study also examined race and ethnicity, using categories of Latino or Hispanic, Black or African American, non-Latino White, and other. We coded the category “other” to represent clients identifying as American Indian, Asian, or another race and ethnicity because our data did not have sufficient clients to analyze these groups separately. Clients also reported demographic and socioeconomic variables including age, education (completing high school), Medi-Cal eligibility, veteran status, and referral source.

### Statistical analyses

Statistical analyses were run using R statistical software to address each aim [35]. Specifically, group comparisons were used to study patterns and disparities in EDT. For instance, t-tests were used to determine if men and women have different average EDTs for each service type. A similar approach was used to compare age groups, Medi-Cal clients, and other study covariates. We constructed generalized linear models to assess the significance of the association between study covariates and EDT while adjusting for potential confounders. Transformations were applied to the dependent variable (EDT) to minimize violations of linear regression assumptions.

For the study’s second aim, multiple statistical techniques were employed to examine the association between EDT and treatment outcomes. To ensure that EDTs did not convey false precision, we estimated a secondary categorical variable for EDT: less than 10 min, between 10 and 20 min, between 20 and 30 min, and more than 30 min. Then, we constructed a logistic regression model to examine the association between EDT categories and odds of completing treatment. The multivariable logistic regression model allowed for isolating the effects of EDT and potential interactions with study covariates. A complete model was first constructed, then models were constructed in an explorative manner such that variables and interactions were iteratively added and assessed for significance.

The data studied in this analysis had an average missing rate of less than 10%. Observations were used whenever the variables of interest in the analysis step were available (pairwise deletion) to maximize the sample size in each analysis step. Missing data analysis was conducted to ensure the randomness of missing data through several packages in R statistical software [35].

## Results

### Aim 1: EDT for subpopulations in Los Angeles County

The average EDT for all episodes was 11.32 min (95% CI = 11.21, 11.43). EDTs were notably higher for counseling services (15.68 min; CI = 15.22, 16.13) than methadone services (10.74 min; CI =10.64, 10.85). No significant difference were found between Google Maps Distance Matrix API and ESRI road network solutions in a randomly selected subsample of 100 observations. Table 1 summarizes EDTs and distribution across time categories. More episodes involving methadone services had an EDT of 10 min or less (53.5%), compared to a third of episodes involving counseling services (33.9%).

**Table 1:** Summary statistics of driving data

	All Episodes (*N* = 22,587)	Counseling (*n* = 2646)	Methadone (*n* = 19,941)
	n (%) or M (95% CI)	n (%) or M (95% CI)	n (%) or M (95% CI)
Estimated drive time			
Average time (Mins)	11.32 (11.21, 11.43)	15.68 (15.22, 16.13)	10.74 (10.64, 10.85)
10 mins or less	11,566 (51.2)	898 (33.9)	10,668 (53.5)
10–20 min	8576 (38.0)	1049 (39.6)	7527 (37.7)
More than 20 mins	2445 (10.8)	699 (26.4)	1746 (8.8)
Estimated drive distance			
Average distance (miles)	11.32 (11.21, 11.43)	15.68 (15.22, 16.13)	10.74 (10.64, 10.85)
1 mile or less	1447 (6.4)	178 (6.7)	1269 (6.4)
1–2 miles	3685 (16.3)	171 (6.5)	3514 (17.6)
3–5 miles	6579 (29.1)	643 (24.3)	5936 (29.8)
6–10 miles	6515 (28.8)	677 (25.6)	5838 (29.3)
More than 10 miles	4361 (19.3)	977 (36.9)	3384 (17.0)
Proportion of additional driving time due to traffic			
Average %	0.15 (0.15, 0.15)	0.13 (0.13, 0.14)	0.15 (0.15, 0.15)
5% or less	3612 (16.40)	378 (14.53)	3234 (16.65)
6–15%	8605 (39.1)	1297 (49.9)	7308 (37.6)
16–25%	6305 (28.6)	655 (25.2)	5650 (29.1)
More than 25%	3499 (15.9)	271 (10.4)	3228 (16.6)
Gender			
Female	7425 (32.9%)	1012 (38.2%)	6413 (32.2%)
Male	15,160 (67.1%)	1634 (61.8%)	13,526 (67.8%)
MediCal Eligible			
Yes	13,094 (57.97%)	698 (26.38%)	12,396 (62.16%)
No	9493 (42.03%)	1948 (73.62%)	7545 (37.84%)
Age Group			
Younger than 25	1524 (7.02%)	339 (15.58%)	1185 (6.06%)
25–34	5368 (24.72%)	845 (38.83%)	4523 (23.15%)
35–45	4193 (19.31%)	488 (22.43%)	3705 (18.96%)
45–54	5338 (24.58%)	340 (15.62%)	4998 (25.58%)
55–64	4292 (19.76%)	159 (7.31%)	4133 (21.15%)
65 and older	1002 (4.61%)	5 (0.23%)	997 (5.10%)
Race			
White	9619 (42.59%)	1305 (49.32%)	8314 (41.69%)
Black	2656 (11.76%)	155 (5.86%)	2501 (12.54%)
Latino	9145 (40.49%)	906 (34.24%)	8239 (41.32%)
Other	955 (4.23%)	172 (6.50%)	783 (3.93%)
Education			
Completed HS	6195 (70.17%)	782 (70.77%)	5413 (70.09%)
Did Not Complete HS	2633 (29.83%)	323 (29.23%)	2310 (29.91%)
Veteran			
Yes	807 (3.57%)	52 (1.97%)	755 (3.79%)
No	21,778 (96.43%)	2594 (98.03%)	19,184 (96.21%)
Referral Source			
Self Referral	20,349 (90.09%)	991 (37.45%)	19,358 (97.08%)
Court Referral	1159 (5.13%)	1079 (40.78%)	80 (0.40%)
Other Source	1079 (4.78%)	576 (21.77%)	503 (2.52%)

### Geographic disparities

**Fig. 1:**
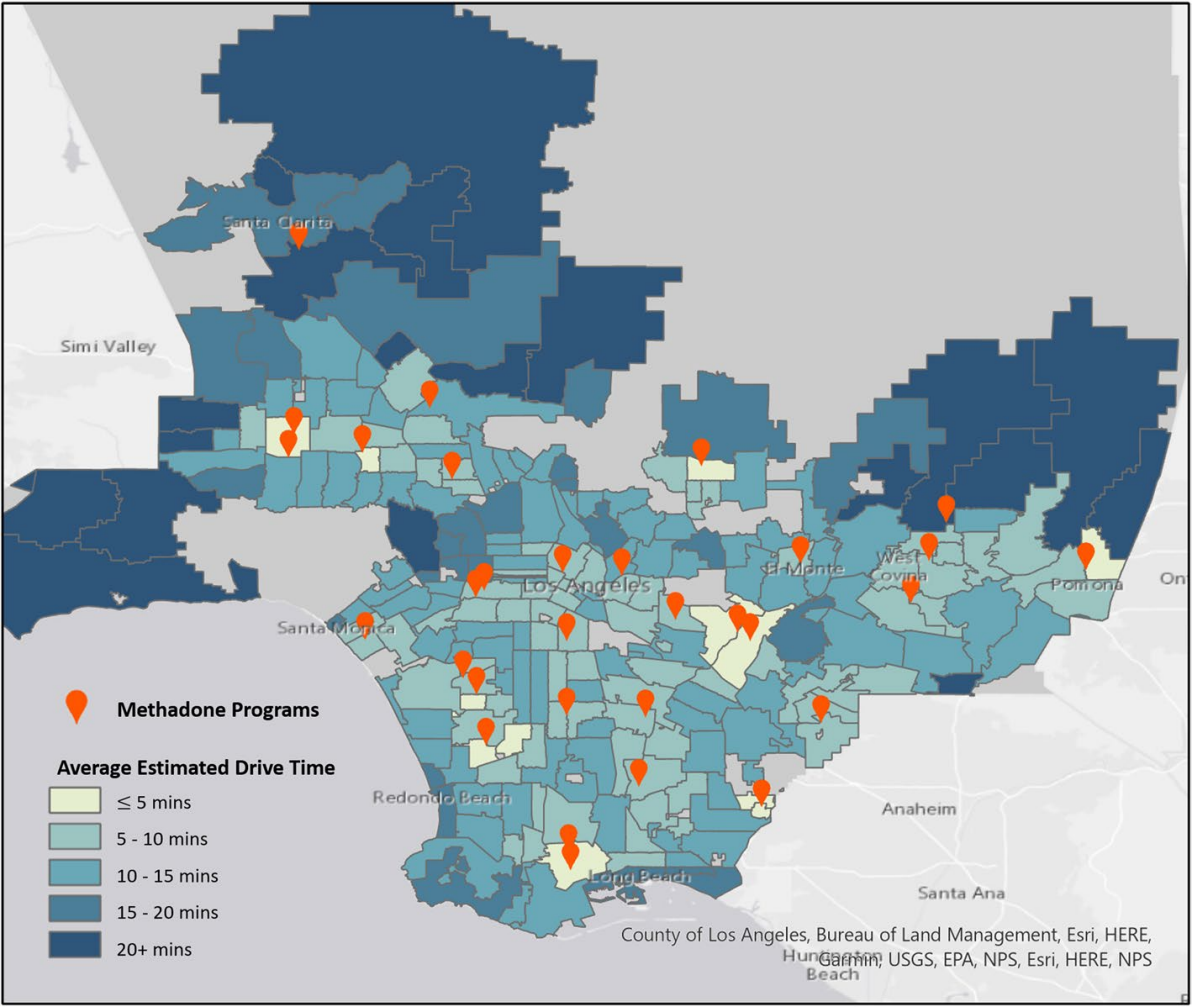
Average Estimated Drive Time to Reach Methadone Programs in Los Angeles by ZIP Code. A combination of GIS and Google Maps API was used to calculate the average time (in minutes) it would take for clients from Los Angeles County ZIP code in the study sample to drive to the methadone programs they attended. For robustness, only ZIP codes with at least 10 episodes are shown

The map of average EDT for each client ZIP code (Fig. 1) reveals wide variation in average EDT to medication-assisted treatment. Several ZIP codes surrounding methadone programs (shown in red) had higher-than-average EDT. This suggests potential quality or access issues regarding the nearest methadone services provider.

### Group disparities

EDT varied across subgroups and services. Specifically, Table 2 highlights the unadjusted differences in EDT for episodes across age groups, gender, race, Medi-Cal eligibility, education, and referral source. For counseling episodes, Medi-Cal-ineligible clients, young adults, White clients, and self-referred clients had a statistically longer EDT than their counterparts (Student’s t-test; *p* < .05) statistically. In methadone episodes, male, Medi-Cal ineligible, young adult, White, and high school-completing clients had statistically longer EDT than their counterparts (Student’s t-test; *p* < .05).

**Table 2:** Comparison of driving times across subgroups

	All Episodes (*N* = 22,587)	Counseling (*n* = 2646)	Methadone (*n* = 19,941)
	M (95% CI)	M (95% CI)	M (95% CI)
Driving time (minutes)	11.32 (11.21, 11.43)	15.68 (15.22, 16.13)	10.74 (10.64, 10.85)
Gender			
Female	11.26 (11.07, 11.45)	15.87 (15.13, 16.62)	10.53 (10.36, 10.71)
Male	11.35 (11.22, 11.48)	15.55 (14.98, 16.13)	10.84 (10.71, 10.97)
Medi-Cal eligible			
Yes	10.63 (10.50, 10.76)	12.14 (11.52, 12.76)	10.55 (10.41, 10.68)
No	12.27 (12.09, 12.46)	16.94 (16.38, 17.51)	11.07 (10.89, 11.24)
Age group			
Younger than 25	12.90 (12.42, 13.37)	16.03 (14.80, 17.26)	12.00 (11.52, 12.48)
25–34	12.24 (11.99, 12.50)	15.50 (14.66, 16.34)	11.63 (11.38, 11.89)
35–44	11.20 (10.96, 11.44)	16.17 (15.20, 17.14)	10.55 (10.32, 10.78)
45–54	10.91 (10.69, 11.13)	16.69 (15.10, 18.29)	10.51 (10.31, 10.72)
55–64	10.07 (9.88, 10.27)	13.52 (12.06, 14.98)	9.94 (9.75, 10.13)
65 or older	10.47 (10.02, 10.91)	12.57 (7.80, 17.33)	10.46 (10.01, 10.90)
Race and ethnicity			
White	12.30 (12.12, 12.49)	16.79 (16.08, 17.50)	11.60 (11.42, 11.78)
Black	10.06 (9.82, 10.30)	17.48 (15.33, 19.63)	9.60 (9.40, 9.81)
Latino	10.50 (10.35, 10.66)	13.82 (13.16, 14.47)	10.14 (9.98, 10.29)
Other	12.04 (11.51, 12.57)	15.80 (14.35, 17.25)	11.21 (10.66, 11.76)
Education			
Completed high school	12.24 (12.01, 12.46)	17.01 (16.09, 17.93)	11.55 (11.33, 11.76)
Did not complete high school	11.16 (10.84, 11.48)	15.18 (14.03, 16.33)	10.60 (10.28, 10.91)
Referral source			
Self	11.05 (10.94, 11.16)	17.36 (16.56, 18.16)	10.73 (10.62, 10.83)
Court	15.49 (14.82, 16.15)	15.89 (15.18, 16.59)	10.05 (8.71, 11.40)
Other	11.92 (11.37, 12.48)	12.39 (11.60, 13.18)	11.40 (10.63, 12.16)

A linear regression (Table 3) fit on EDT (log transformed) for each service type revealed strong associations of Medi-Cal eligibility and referral source with EDT in counseling episodes. The directionality of the association from the regression agreed with findings from the group analysis on counseling episodes. In methadone episodes, significant associations emerged between younger age, White race, male gender, and high school completion and longer EDT. The regression models allowed for an adjusted assessment of covariates and their association with EDT. The fit measures of the regression models were expectedly low (R^2^ < .10) due to estimating a continuous outcome variable (log EDT) from categorical variables.

**Table 3:** Log estimated driving time (EDT) for counseling and methadone episodes

	Dependent Variable	
	Log EDT	
	Counseling	Methadone
	β (95% CI)	β (95% CI)
Age group		
Younger than 25	(Reference)	(Reference)
25–34	1.029 (0.867, 1.220)	0.933 (0.869, 1.000)
35–44	1.033 (0.861, 1.240)	0.871*** (0.810, 0.938)
45–54	0.969 (0.803, 1.170)	0.934 (0.869, 1.003)
55–64	0.941 (0.736, 1.203)	0.848*** (0.787, 0.915)
65 or older	0.704 (0.323, 1.533)	0.892* (0.806, 0.986)
Medi-Cal eligible	0.856* (0.754, 0.972)	(0.943, 1.013)
Race and ethnicity		
White	(Reference)	(Reference)
Black	1.004 (0.829, 1.216)	0.953 (0.904, 1.005)
Latino	0.616 (0.311, 1.220)	1.050 (0.647, 1.702)
Other	0.878 (0.685, 1.126)	0.909 (0.825, 1.000)
Veteran	0.862 (0.597, 1.246)	1.045 (0.958, 1.139)
Referral source		
Self	(Reference)	(Reference)
Court	0.859* (0.758, 0.974)	0.942 (0.629, 1.412)
Other	0.586*** (0.503, 0.682)	1.069 (0.961, 1.189)
Female	0.938 (0.837, 1.052)	0.952** (0.917, 0.988)
Completed high school	1.067 (0.936, 1.215)	1.097*** (1.054, 1.141)
Constant	12.588*** (9.651, 16.419)	(5.551, 12.641)
Observations	791	5561
R^2^	0.078	0.014
Adjusted R^2^	0.061	0.012
Residual std. error	0.771 (df = 776)	0.651 (df = 5546)
F	4.696*** (df = 14; 776)	5.765*** (df = 14; 5546)

*p < .05. **p < .01. ***p < .001

Linear regression models on the log estimated driving time (EDT) as a function of client attributes by service type (counseling and methadone)

### Association between age and EDT for methadone treatment

Episodes involving older clients were found to be associated with shorter EDT in group comparisons and the regression model. Individuals between 35 and 44 years old had EDTs to care that were 12.9% shorter than those younger than 25. For individuals aged 55 or older, their EDTs were approximately 15% shorter than those younger than 25, after adjusting for potential confounders.

### Association between gender and EDT for methadone treatment

In the linear regression model, female clients had an EDT that was 4.8% shorter (95% CI = 1.2, 8.3%) than male clients after adjusting for potential confounders. The same difference was observed in the group differences shown in Table 2, with a statistically significant difference in average EDT between male and female clients (*p* < .05).

### Association between high school completion and EDT for methadone treatment

Episodes involving clients who completed high school had 9.7% longer EDT ((95% CI: 5.4, 14.1%) to methadone treatment after adjusting for potential confounders. A consistent and statistically significant difference was observed in the group comparisons based on high school completion shown in Table 2.

### Aim 2: association between EDT and treatment completion

On average, 10.88% (CI = 10.26, 11.51%) of episodes resulted in completion of treatment goals. The completion rate of counseling episodes was 23.6% (CI = 21.6, 25.6%), whereas the completion rate for methadone was notable lower, at 8.1% (CI = 7.49, 8.70%). Lower episode completion was found to be associated with longer EDTs for methadone episodes. Treatment plan completion rates for each subgroup and service type are summarized in appendix 1. In the logistic regression model fit on the completion variable for each episode, longer EDT was significantly associated with a lower probability of completion for methadone episodes after adjusting for potential confounders (Table 4). For robustness, mixed effects models with a random intercept for the number of client-level prior episodes were also constructed. Coefficient values and fit measures did not change in mixed effects models, but the random intercept for episode history had non-zero variance.

**Table 4:** Logistic regression model with treatment completion

	Dependent Variable	
	Episode Completion (1 = complete, 0 = incomplete)	
	Counseling	Methadone
	OR (95% CI)	OR (95% CI)
Estimated driving time		
10 mins or less	(Reference)	(Reference)
11–20 min	0.960 (0.577, 1.595)	0.670** (0.509, 0.878)
21–30 min	0.839 (0.449, 1.538)	0.594* (0.357, 0.943)
More than 30 mins	0.736 (0.341, 1.520)	1.010 (0.455, 2.000)
Age group		
Younger than 25	(Reference)	(Reference)
25–34	0.828 (0.435, 1.597)	1.013 (0.637, 1.657)
35–44	0.781 (0.393, 1.561)	1.341 (0.826, 2.230)
45–54	0.915 (0.441, 1.901)	1.477 (0.919, 2.439)
55–64	1.252 (0.499, 3.090)	1.219 (0.714, 2.114)
65 or older	–	0.573 (0.186, 1.459)
Medi-Cal eligible	0.576* (0.354, 0.920)	0.337*** (0.251, 0.448)
Race and ethnicity		
White	(Reference)	(Reference)
Black	1.451 (0.698, 2.945)	0.966 (0.607, 1.494)
Black	1.947 (0.073, 52.027)	2.134 (0.106, 15.411)
Other	1.402 (0.564, 3.298)	1.351 (0.701, 2.413)
Veteran	0.840 (0.164, 3.492)	0.889 (0.390, 1.765)
Referral source		
Self	(Reference)	(Reference)
Court	1.984** (1.220, 3.275)	–
Other	1.520 (0.799, 2.869)	1.438 (0.619, 2.933)
Female	1.247 (0.798, 1.944)	0.956 (0.721, 1.258)
Completed high school	2.091** (1.288, 3.471)	1.221 (0.899, 1.682)
Constant	0.722 (0.252, 2.015)	0 (− 643, 2,454,803)
Observations	462	2888
Log likelihood	−264.566	− 879.704
Akaike information criterion	565.133	1795.407

*p < .05. **p < .01. ***p < .001

### Association between EDT and completion of methadone treatment plan

According to the logistic regression findings in Table 4, an EDT between 10 and 20 min was associated with a 37% reduction in the probability of completing a methadone episode compared to an EDT of less than 10 min, after adjusting for potential confounders. Moreover, the probability of completing a methadone episode was 40.6% lower if the EDT was between 20 and 30 min, compared to an EDT of less than 10 min. We did not find a significant relationship between EDT and counseling episode completion.

## Discussion

The analysis revealed significant disparities in EDT and a strong relationship with OUD treatment plan completion. We found disparities in EDT to OUD medication treatment across age, gender, and socioeconomic status (SES). Longer EDTs were also associated with a significant reduction in the odds of completing methadone treatment plans. These findings were consistent across several modes of analysis, supporting the robustness of the findings.

Maps of episode EDTs by ZIP code show visible disparities in Central Los Angeles, where quality or access issues may persist despite proximity to treatment facilities. In group comparisons of EDT among strata of Los Angeles County (Aim 1), we found disparities in average EDT across gender, race and ethnicity, age, and SES groups. The log-transformed linear regression confirmed the gender, age, and SES associations with EDT for methadone, with young, male, and higher-SES clients having longer EDT to methadone treatment.

Shorter commutes to treatment for at-risk groups (female, ethnic minorities, and lower SES clients) have been observed and confirmed in the literature. For example, Hyder et al. (2021) found that Black SUD clients are less likely to live in OUD treatment deserts than their White counterparts [7]. Nevertheless, they are more likely to make more frequent trips to receive care due to their greater likelihood of being prescribed methadone rather than office-based buprenorphine. Similarly, although Rosenblum et al. (2011) found that ethnoracial minorities traveled shorter distances on average to reach OUD treatment than White patients, they noted that “travel distance can suggest more resources to travel a greater distance,” meaning travel distance can be “both a sign of privilege and at the same time a burden and risk factor for treatment dropout” [36]. Additionally, low-income, non-White, and foreign-born persons are all significantly more likely to rely on public transit for their transportation needs. They may face a time penalty that is not reflected in the single-mode calculations made in this paper [37–39].

Counseling episodes involved significantly longer EDT than methadone episodes (by 4.94 min; *p* < .001). We found a difference in EDT for counseling episodes based on Medi-Cal eligibility and referral source. Medi-Cal-eligible clients had shorter commutes (found in both modes of analysis). Self-referred clients were estimated to commute longer compared to those with a court referral or other referral sources. However, differences in EDT were not associated with changes in the odds of completing counseling treatment plans, as observed for methadone episodes. Albeit conjectural, this finding may show that methadone programs are more likely to be closer to minority communities whose residents may be more likely to be referred to treatment by the court system. Additionally, the fewer trips required for counseling episodes reduce the influence of EDT on treatment completion, unlike methadone episodes, which may require frequent visits.

In the completion analysis (Aim 2), we found that episode treatment plan completion was closely associated with EDT for methadone episodes. Methadone treatment completion significantly dropped for clients with longer EDT. In the logistic regression model examining completion among methadone episodes, we found that an EDT of 10 to 20 min was associated with a 33% (CI = 12.2, 40.1%) drop in the odds of completing a methadone episode compared to an episode where the client had an EDT of less than 10 min. Additionally, an EDT of 20 to 30 min was associated with a 40.6% (CI = 5.7, 64.3%) drop in the odds of completing a methadone episode compared to an episode where the client had an EDT of less than 10 min. The critical association measured in this study is central to improving access and guiding further studies in understanding factors that influence travel time. More specifically, this can help us understand factors such as driving farther due to availability of client resources (with hypothesized improved outcomes) or longer driving time due to lack of access. The above relationship holds for 96.8% of the study sample, but the relationship between longer EDT and completion did not appear for driving times beyond 30 min in the regression. This may potentially be explained by the small number of client episodes falling in this category (~ 3%).

One study assumption, using a single mode of transportation, may have influenced the findings for vulnerable groups. However, studies have shown that the single-mode approach has been found to predict a similar pattern of health care accessibility compared to multimodal approaches, with a high correlation observed between single-mode and multimodal accessibility rates [25]. Additionally, if transportation mode data for episodes or clients are available, the methods used are amicable to integrating such diverse travel modes into the analysis.

## Conclusion

Using advanced GIS and geospatial methods to calculate EDT, we scalably supplemented administrative data with commuting variables. Specifically, we handled a dataset with more than 22,000 episodes and captured drive-time features along with expected traffic contributions for each episode. The average EDT was longer than 10 min to methadone treatment and 15 min to outpatient counseling, signaling an untapped opportunity to improve completion and initiation by enhancing spatial access to treatment programs.

By improving access to treatment programs and reducing the need for longer driving time to methadone treatment, our findings suggest a likely increase to treatment completion rates. More specifically, 46.5% of the methadone treatment episodes had an EDT longer than 10 min, and these episodes could have a 33% increase in the probability of completing the treatment plan if access to treatment programs is improved such that driving times are less than 10 min. This significant expected improvement is likely to produce a meaningful change given the imperative for timely access to treatment amid the insidious opioid epidemic affecting the United States.

Previous studies have shown a lack of stability in treatment facilities in Los Angeles County, with 62% of programs closing in a 5-year period [40]. Although methadone programs were found to be more stable, the frequency of closures can be tied to significant changes in EDT to treatment. As programs close or relocate, clients’ EDT to treatment varies, potentially leading to avoidable variability in treatment completion and continuity. Therefore, it is important to investigate the link between robust treatment systems (fewer closures) and high and stable treatment completion rates mediated through stable EDTs.

Additionally, the findings support more targeted investigations involving at-risk groups to understand differences in EDT to methadone treatment. Female, older, and lower-SES clients had shorter commuting times. Nevertheless, lower-SES clients still had lower completion rates when adjusting for EDT and other covariates. Therefore, it is vital to investigate the mechanisms and factors influencing EDT (e.g., access, availability of resources, quality) and how they tie to completion of treatment plans.

This study contributed to the growing body of literature on spatial travel barriers by documenting driving times by car to OUD treatment facilities in Los Angeles County, a large and highly diverse car-centric metropolis, and its association with the completion of treatment plans. With these data, future studies will be better equipped to design analyses that explore the degree and type of influence of travel distance and time on treatment outcomes, especially in vulnerable populations that face more significant health care disparities overall.

## Supplementary Information

**Additional file 1.**

## Data Availability

The data that support the findings of this study are available from the City of Los Angeles Department of Public Health – Substance Abuse Prevention and Control but restrictions apply to the availability of these data, which were used under license for the current study, and so are not publicly available. Data are however available from the authors upon reasonable request and with permission of City of Los Angeles Department of Public Health – Substance Abuse Prevention and Control. Distance related data used to supplement previously descrived data is are available in the Google Developer Services with the Distance Matrix API, https://developers.google.com/maps/documentation/distance-matrix/overview.

## References

[1] Centers for Disease Control and Prevention (2021). Drug overdose deaths in the U.S. top 100,000 annually.

[2] Srivastava A, Kahan M, Nader M (2017). Primary care management of opioid use disorders: abstinence, methadone, or buprenorphine-naloxone?. Can Fam Physician.

[3] Goedel WC, Shapiro A, Cerdá M, Tsai JW, Hadland SE, Marshall BDL (2020). Association of Racial/ethnic segregation with treatment capacity for opioid use disorder in counties in the United States. JAMA Netw Open.

[4] Lagisetty PA, Ross R, Bohnert A, Clay M, Maust DT (2019). Buprenorphine treatment divide by race/ethnicity and payment. JAMA Psychiatry.

[5] National Institute on Drug Abuse (2019). Access to addiction services differs by race and gender.

[6] Substance Abuse and Mental Health Services Administration (2020). The opioid crisis and the black/African American population: an urgent issue.

[7] Hyder A, Lee J, Dundon A, Southerland LT, All D, Hammond G (2021). Opioid treatment deserts: concept development and application in a US Midwestern urban county. Plos One.

[8] Ogneva-Himmelberger Y. Spatial Analysis of Drug Poisoning Deaths and Access to Substance-use Disorder Treatment in the United States. In: Proceedings of the 5th International Conference on Geographical Information Systems Theory, Applications and Management - HGIS. Heraklion: SciTePress; 2019. p. 315–21.

[9] Perron BE, Gillespie DF, Alexander-Eitzman B, Delva J (2010). Availability of outpatient substance use disorder treatment programs in the United States. Subst Use Misuse.

[10] Guerrero EG, Kao D, Perron BE (2013). Travel distance to outpatient substance use disorder treatment facilities for Spanish-speaking clients. Int J Drug Policy..

[11] Beardsley K, Wish ED, Fitzelle DB, O’Grady K, Arria AM (2003). Distance traveled to outpatient drug treatment and client retention. J Subst Abus Treat.

[12] Kleinman RA (2020). Comparison of driving times to opioid treatment programs and pharmacies in the US. JAMA Psychiatry.

[13] Amiri S, Hirchak K, McDonell MG, Denney JT, Buchwald D, Amram O (2021). Access to medication-assisted treatment in the United States: comparison of travel time to opioid treatment programs and office-based buprenorphine treatment. Drug Alcohol Depend.

[14] Amiri S, Lutz RB, McDonell MG, Roll JM, Amram O (2020). Spatial access to opioid treatment program and alcohol and cannabis outlets: analysis of missed doses of methadone during the first, second, and third 90 days of treatment. Am J Drug Alcohol Abuse.

[15] Klinger JL, Karriker-Jaffe KJ, Witbrodt J, Kaskutas LA (2018). Effects of distance to treatment on subsequent alcohol consumption. Drugs Educ Prev Policy.

[16] Barbosa H, Hazarie S, Dickinson B, Bassolas A, Frank A, Kautz H (2021). Uncovering the socioeconomic facets of human mobility. Sci Rep.

[17] Costa C, Ha J, Lee S (2021). Spatial disparity of income-weighted accessibility in Brazilian cities: application of a Google maps API. J Transp Geogr.

[18] Dėdelė A, Miškinytė A, Andrušaitytė S, Nemaniūtė-Gužienė J (2020). Dependence between travel distance, individual socioeconomic and health-related characteristics, and the choice of the travel mode: a cross-sectional study for Kaunas, Lithuania. J Transp Geogr.

[19] Graves BA (2008). Integrative literature review: a review of literature related to geographical information systems, healthcare access, and health outcomes. Perspect Health Inf Manag AHIMA Am Health Inf Manag Assoc.

[20] Kelly C, Hulme C, Farragher T, Clarke G (2016). Are differences in travel time or distance to healthcare for adults in global north countries associated with an impact on health outcomes? A systematic review. BMJ Open.

[21] Qi Z, Lim S, Hossein RT (2020). Assessment of transport equity to central Business District (CBD) in Sydney. Australia Transp Lett.

[22] County Of Los Angeles Enterprise GIS. 2010 Census data by block, vol. 2010: LA County eGIS Program. https://egis-lacounty.hub.arcgis.com/datasets/lacounty::2010-census-data-by-block/about. Accessed 1 Nov 2021

[23] Google Developers (2021). Google maps distance matrix API. Google Maps Platform.

[24] ESRI. Create Drive-Time Areas. ArcGIS Online Help | Documentation. https://doc.arcgis.com/en/arcgis-online/analyze/create-drive-time-areas.htm. Accessed 8 Feb 2022.

[25] Mao L, Nekorchuk D (2013). Measuring spatial accessibility to healthcare for populations with multiple transportation modes. Health Place.

[26] Substance Abuse and Mental Health (2019). Treatment episode data set (TEDS): 2017. Admissions to and discharges from publicly-funded substance use treatment.

[27] Guerrero EG, Campos M, Urada D, Yang JC (2012). Do cultural and linguistic competence matter in Latinos’ completion of mandated substance abuse treatment?. Subst Abuse Treat Prev Policy.

[28] Guerrero EG, Cepeda A, Duan L, Kim T (2012). Disparities in completion of substance abuse treatment among Latino subgroups in Los Angeles County, CA. Addict Behav.

[29] Guerrero EG, Marsh JC, Duan L, Oh C, Perron B, Lee B (2013). Disparities in completion of substance abuse treatment between and within racial and ethnic groups. Health Serv Res.

[30] Arndt S, Acion L, White K (2013). How the states stack up: disparities in substance abuse outpatient treatment completion rates for minorities. Drug Alcohol Depend.

[31] Askari MS, Martins SS, Mauro PM (2020). Medication for opioid use disorder treatment and specialty outpatient substance use treatment outcomes: differences in retention and completion among opioid-related discharges in 2016. J Subst Abus Treat.

[32] Mennis J, Stahler GJ, El Magd SA, Baron DA (2019). How long does it take to complete outpatient substance use disorder treatment? Disparities among blacks, Hispanics, and whites in the US. Addict Behav.

[33] Stahler GJ, Mennis J (2018). Treatment outcome disparities for opioid users: are there racial and ethnic differences in treatment completion across large US metropolitan areas?. Drug Alcohol Depend.

[34] Substance Abuse and Mental Health Services Administration (2009). The TEDS report: predictors of substance abuse treatment completion or transfer to further treatment, by service type.

[35] R Core Team (2021). R: A language and environment for statistical computing.

[36] Rosenblum A, Cleland CM, Fong C, Kayman DJ, Tempalski B, Parrino M (2011). Distance traveled and cross-state commuting to opioid treatment programs in the United States. J Environ Public Health.

[37] National Equity Atlas (2019). Car access: everyone needs reliable transportation access and in most American communities that means a car.

[38] Neighborhoods organizing for change, TakeAction Minnesota, ISAIAH, the Center for Popular Democracy (2015). It’s about time: the transit time penalty and its racial implications undefined.

[39] U.S. Census Bureau (2021). Commuting by public transportation in the United States: 2019.

[40] Guerrero EG, Alibrahim A, Howard DL, Wu S, D’Aunno T (2020). Stability in a large drug treatment system: examining the role of program size and performance on service discontinuation. Int J Drug Policy.

