# Correction to: Disparities in expected driving time to opioid treatment and treatment completion: findings from an exploratory study

**DOI:** 10.1186/s12913-022-07993-5

**Published:** 2022-05-09

**Authors:** Abdullah Alibrahim, Jeanne C. Marsh, Hortensia Amaro, Yinfei Kong, Tenie Khachikian, Erick Guerrero

**Affiliations:** 1 Industrial & Management Systems Engineering, College of Engineering & Petroleum, Kuwait University, Kuwait, Kuwait; 2 Geo-Health Lab, Dasman Diabetes Institute, Kuwait, Kuwait; 3 Crown Family School of Social Work, Policy, and Practice, University of Chicago, Chicago, USA; 4 Robert Stempel College of Public Health and Social Work and Herbert Wertheim College of Medicine, Florida Internation University, Miami, USA; 5 College of Business and Economics, California State University Fullerton, Fullerton, USA; 6 I-Lead Institute, Research to End Healthcare Disparities Corp, Los Angeles, USA

**Correction to: BMC Health Serv Res 22, 478 (2022)**

**https://doi.org/10.1186/s12913-022-07886-7**

Following publication of the original article [1], the authors identified an error in the third sentence in the Results section of the Abstract.

The sentence originally read:

Young, male, **less** formally educated, and Medi-Cal-ineligible clients drove longer to treatment.

The sentence should read:

Young, male, **more** formally educated, and Medi-Cal-ineligible clients drove longer to treatment.

The original article [1] has been corrected.
