# Supplementary material for: Disparities in expected driving time to opioid treatment and treatment completion: findings from an exploratory study
Source: BMC Health Serv Res. 2022 Apr 11;22:478. doi: 10.1186/s12913-022-07886-7 (PMC8996398; doi:10.1186/s12913-022-07886-7)
Supplement: Supplementary file 1 — Additional file 1. [file 12913_2022_7886_MOESM1_ESM.docx]

Appendix 1. Completion rates for counseling and methadone episodes based on estimated driving time by study covariates

|  |  | Counseling | | | | Methadone | | | |
| --- | --- | --- | --- | --- | --- | --- | --- | --- | --- |
|  | Full Sample  (N = 9,653) | 10 Mins or Less  (n = 608) | 11–20 Mins  (n = 685) | 21–30 mins  (n = 300) | 30+ Mins  (n = 141) | 10 Mins or Less  (n = 4,009) | 11–20 Mins  (n = 3,031) | 21–30 mins  (n = 627) | 30+ Mins  (n = 252) |
| **Completion rate** |  |  |  |  |  |  |  |  |  |
| All | 0.11 (0.10, 0.12) | 0.23 (0.20, 0.27) | 0.25 (0.22, 0.29) | 0.22 (0.18, 0.27) | 0.17 (0.11, 0.24) | 0.09 (0.08, 0.10) | 0.07 (0.06, 0.08) | 0.08 (0.06, 0.10) | 0.10 (0.06, 0.14) |
| **Gender** |  |  |  |  |  |  |  |  |  |
| Female | 0.11 (0.10, 0.12) | 0.30 (0.23, 0.36) | 0.26 (0.21, 0.32) | 0.19 (0.12, 0.27) | 0.21 (0.09, 0.34) | 0.08 (0.07, 0.10) | 0.06 (0.05, 0.08) | 0.08 (0.04, 0.11) | 0.11 (0.02, 0.19) |
| Male | 0.11 (0.10, 0.12) | 0.21 (0.17, 0.25) | 0.25 (0.21, 0.29) | 0.24 (0.18, 0.30) | 0.15 (0.08, 0.23) | 0.09 (0.08, 0.10) | 0.07 (0.06, 0.08) | 0.08 (0.05, 0.10) | 0.10 (0.06, 0.14) |
| **Medi-Cal eligible** |  |  |  |  |  |  |  |  |  |
| Yes | 0.06 (0.06, 0.07) | 0.22 (0.17, 0.28) | 0.16 (0.11, 0.22) | 0.23 (0.11, 0.36) | 0.14 (-0.01, 0.30) | 0.05 (0.04, 0.06) | 0.04 (0.03, 0.05) | 0.04 (0.02, 0.07) | 0.05 (0.01, 0.09) |
| No | 0.16 (0.15, 0.17) | 0.24 (0.20, 0.28) | 0.29 (0.25, 0.33) | 0.22 (0.17, 0.27) | 0.18 (0.11, 0.25) | 0.14 (0.12, 0.16) | 0.10 (0.08, 0.11) | 0.12 (0.08, 0.15) | 0.17 (0.09, 0.24) |
| **Age group** |  |  |  |  |  |  |  |  |  |
| Younger than 25 | 0.11 (0.09, 0.14) | 0.26 (0.16, 0.37) | 0.18 (0.08, 0.27) | 0.20 (0.08, 0.33) | 0.18 (-0.01, 0.36) | 0.09 (0.06, 0.12) | 0.07 (0.04, 0.11) | 0.09 (0.03, 0.15) | 0.07 (-0.02, 0.16) |
| 25–34 | 0.10 (0.09, 0.11) | 0.17 (0.11, 0.22) | 0.23 (0.17, 0.29) | 0.20 (0.11, 0.28) | 0.22 (0.10, 0.34) | 0.08 (0.06, 0.10) | 0.05 (0.04, 0.07) | 0.08 (0.04, 0.12) | 0.08 (0.02, 0.14) |
| 35–44 | 0.11 (0.10, 0.13) | 0.25 (0.16, 0.34) | 0.32 (0.24, 0.40) | 0.23 (0.12, 0.35) | 0.12 (-0.04, 0.29) | 0.09 (0.07, 0.11) | 0.07 (0.05, 0.09) | 0.10 (0.05, 0.16) | 0.11 (0.02, 0.20) |
| 45–54 | 0.09 (0.07, 0.10) | 0.32 (0.21, 0.43) | 0.23 (0.12, 0.33) | 0.28 (0.10, 0.46) | 0.12 (-0.04, 0.28) | 0.07 (0.05, 0.09) | 0.07 (0.05, 0.09) | 0.05 (0.01, 0.09) | 0.10 (0.01, 0.20) |
| 55–64 | 0.10 (0.08, 0.11) | 0.33 (0.19, 0.47) | 0.16 (0.03, 0.28) | 0.53 (0.27, 0.79) | 0.33 (-0.32, 0.99) | 0.08 (0.06, 0.10) | 0.08 (0.06, 0.11) | 0.05 (0.00, 0.11) | 0.04 (-0.04, 0.11) |
| 65 or older | 0.05 (0.03, 0.07) | 0.00 | 0.00 |  |  | 0.07 (0.03, 0.11) | 0.03 (0.00, 0.05) | 0.13 (-0.04, 0.31) | 0.00 (0.00, 0.00) |
| **Race and ethnicity** |  |  |  |  |  |  |  |  |  |
| White | 0.12 (0.11, 0.13) | 0.27 (0.21, 0.32) | 0.30 (0.25, 0.35) | 0.20 (0.14, 0.27) | 0.15 (0.07, 0.22) | 0.10 (0.08, 0.11) | 0.12 (0.11, 0.13) | 0.27 (0.21, 0.32) | 0.30 (0.25, 0.35) |
| Black | 0.08 (0.06, 0.09) | 0.25 (0.09, 0.41) | 0.23 (0.10, 0.36) | 0.35 (0.15, 0.55) | 0.44 (0.19, 0.69) | 0.06 (0.04, 0.09) | 0.08 (0.06, 0.09) | 0.25 (0.09, 0.41) | 0.23 (0.10, 0.36) |
| Latino | 0.10 (0.09, 0.11) | 0.22 (0.16, 0.27) | 0.20 (0.16, 0.25) | 0.24 (0.15, 0.32) | 0.17 (0.03, 0.31) | 0.09 (0.08, 0.10) | 0.10 (0.09, 0.11) | 0.22 (0.16, 0.27) | 0.20 (0.16, 0.25) |
| Other | 0.13 (0.10, 0.16) | 0.18 (0.05, 0.31) | 0.33 (0.19, 0.46) | 0.23 (0.07, 0.40) | 0.00 (0.00, 0.00) | 0.11 (0.06, 0.16) | 0.13 (0.10, 0.16) | 0.18 (0.05, 0.31) | 0.33 (0.19, 0.46) |
| **Education** |  |  |  |  |  |  |  |  |  |
| Completed high school | 0.13 (0.12, 0.14) | 0.28 (0.21, 0.36) | 0.33 (0.25, 0.41) | 0.28 (0.17, 0.38) | 0.29 (0.16, 0.42) | 0.11 (0.10, 0.13) | 0.10 (0.08, 0.11) | 0.08 (0.04, 0.11) | 0.10 (0.04, 0.16) |
| Did not complete high school | 0.10 (0.09, 0.12) | 0.27 (0.18, 0.37) | 0.19 (0.10, 0.27) | 0.25 (0.11, 0.39) | 0.12 (-0.04, 0.29) | 0.10 (0.08, 0.12) | 0.06 (0.04, 0.08) | 0.04 (0.00, 0.08) | 0.12 (-0.01, 0.24) |
| **Veteran** |  |  |  |  |  |  |  |  |  |
| Yes | 0.08 (0.05, 0.11) | 0.30 (0.00, 0.60) | 0.18 (-0.01, 0.36) | 0.50 (-0.07, 1.07) | 0.00 | 0.07 (0.03, 0.11) | 0.03 (0.00, 0.07) | 0.10 (-0.03, 0.22) | 0.17 (-0.16, 0.49) |
| No | 0.11 (0.10, 0.12) | 0.23 (0.20, 0.27) | 0.26 (0.22, 0.29) | 0.22 (0.17, 0.27) | 0.17 (0.11, 0.24) | 0.09 (0.08, 0.10) | 0.07 (0.06, 0.08) | 0.08 (0.05, 0.10) | 0.10 (0.06, 0.14) |
| **Referral source** |  |  |  |  |  |  |  |  |  |
| Self | 0.09 (0.08, 0.09) | 0.20 (0.14, 0.25) | 0.19 (0.14, 0.24) | 0.16 (0.10, 0.23) | 0.10 (0.03, 0.17) | 0.09 (0.08, 0.10) | 0.07 (0.06, 0.08) | 0.08 (0.06, 0.10) | 0.09 (0.05, 0.13) |
| Court | 0.30 (0.26, 0.33) | 0.28 (0.22, 0.34) | 0.32 (0.27, 0.37) | 0.32 (0.23, 0.41) | 0.28 (0.15, 0.41) | 0.07 (-0.07, 0.21) | 0.08 (-0.08, 0.25) | 0.00 | 0.50 (-0.48, 1.48) |
| Other | 0.17 (0.14, 0.21) | 0.22 (0.16, 0.28) | 0.22 (0.15, 0.30) | 0.17 (0.07, 0.26) | 0.17 (-0.01, 0.34) | 0.05 (-0.01, 0.10) | 0.09 (0.01, 0.17) | 0.00 (0.00, 0.00) | 0.25 (-0.07, 0.57) |
